# CCR3 antagonist protects against induced cellular senescence and promotes rejuvenation in periodontal ligament cells for stimulating pulp regeneration in the aged dog

**DOI:** 10.1038/s41598-020-65301-9

**Published:** 2020-05-25

**Authors:** Mohammed Zayed, Koichiro Iohara, Hideto Watanabe, Misako Nakashima

**Affiliations:** 1 Department of Stem Cell Biology and Regenerative Medicine, National Center for Geriatrics and Gerontology, Research Institute, Obu, Aichi, 474-8511 Japan; 2 Department of Animal Surgery, College of Veterinary Medicine, South Valley University, Qena, 83523 Egypt; 3 Institute for Molecular Science of Medicine, Aichi Medical University, Nagakute, Aichi, 480-1195 Japan; 4 Aeras Bio Inc., Air Water Group, Kobe, Hyogo, 650-047 Japan

**Keywords:** Developmental biology, Translational research

## Abstract

Pulp regeneration after transplantation of mobilized dental pulp stem cells (MDPSCs) declines in the aged dogs due in part to the chronic inflammation and/or cellular senescence. Eotaxin-1/C-C motif chemokine 11 (CCL11) is an inflammation marker via chemokine receptor 3 (CCR3). Moreover, CCR3 antagonist (CCR3A) can inhibit CCL11 binding to CCR3 and prevent CCL11/CCR3 signaling. The study aimed to examine the effect of CCR3A on cellular senescence and anti-inflammation/immunomodulation in human periodontal ligament cells (HPDLCs). The rejuvenating effects of CCR3A on neurite extension and migratory activity to promote pulp regeneration in aged dog teeth were also evaluated. *In vivo*, the amount of regenerated pulp tissues was significantly increased by transplantation of MDPSCs with CCR3A compared to control without CCR3A. *In vitro*, senescence of HPDLCs was induced after *p*-Cresol exposure, as indicated by increased cell size, decreased proliferation and increased senescence markers, *p21* and *IL-1β*. Treatment of HPDLCs with CCR3A prevented the senescence effect of *p*-Cresol. Furthermore, CCR3A significantly decreased expression of CCL11, increased expression of immunomodulatory factor, IDO, and enhanced neurite extension and migratory activity. In conclusion, CCR3A protects against *p*-Cresol-induced cellular senescence and enhances rejuvenating effects, suggesting its potential utility to stimulate pulp regeneration in the aged teeth.

## Introduction

Optimal oral condition is essential to enhance health state and attendant psychological effects in the elder society^1,2^. The pulp/dentin regenerative therapy by dental pulp stem cells (DPSCs) is a promising approach to preserve the function and endurance of the tooth which leads to optimal oral health condition^3^. Transplanted mesenchymal stem cells (MSCs) have presented outstanding therapeutic efficacy in many preclinical/clinical disease models in terms of their growth and differentiation capabilities and trophic effects including proliferative, migratory, cell survival and immunomodulatory effects^4^. However, it has been stated by several types of research that the regenerative capacity and naïve properties of MSCs to maintain tissue regeneration progressively decline with age and pathological conditions^5,6^. Moreover, several pathological changes such as increased systemic and local inflammation (inflamm-aging), cellular senescence, immune decline, and stem cell dysfunction are associated with aged tissues^7^. Previously, we demonstrated that mobilized dental pulp stem cells (MDPSCs), a subset of DPSCs, have a high pulp regenerative potential^8^. As noted, despite little difference in the *in vitro* properties of MDPSCs between young and aged teeth, the amount of the regenerated pulp tissue in the aged teeth was less in comparison to the young teeth^9^. Aged teeth are characterized by increasing cementum which may cause constriction of the apical region^10^, preventing migration of resident stem cells from the surrounding tissue inside the root canal. Moreover, there is chronic inflammation in the apical region that might decrease pulp regeneration in aged teeth^11^.

Chemokines are a family of cytokines and secreted proteins with chemotactic activity through interaction with G protein-linked transmembrane receptors expressed on the surface of the target cells^12^. Chemokines play an important role in many pathological progressions including the inflammatory process, thereby creating a key pathogenic event for chronic inflammation^13^. Eotaxin-1, encoded by the CCL11 gene, is a chemokine belonging to the CC chemokine family and produced by a variety of cell types including endothelial cells, epithelial cells, eosinophils, fibroblasts, keratinocytes, chondrocytes, and dental pulp cells^14–17^. CCL11 binds to the chemokine receptors CCR2, CCR3, and CCR5, with the highest affinity to CCR3^18,19^. By interacting with CCR3, CCL11 stimulates the migration of mast cells, eosinophils, Th2- cells, basophils, neutrophils, and macrophages^20^ and initiate inflammation. Moreover, high levels of CCL11 have been described in several chronic inflammatory diseases, such as allergic rhinitis^21^, atopic dermatitis^22^, and rheumatoid arthritis^23^. As well as, it has been involved as an aging marker and increased with donor age^24^, CCL11 acts as a biological marker for pulp inflammation via induction of chemotaxis of eosinophils^25^. Thus, inhibition of the chemokine system, either at the ligand or at the receptor level, is a potential treatment for enhancement of pulp regeneration in the aged teeth. Therefore, we hypothesized that CCR3 antagonist (CCR3A) may improve chronic inflammation and pulp regeneration in aged teeth. The application of MSCs together with some factors, such as cytokines and/or biometrics could establish a healthy paracrine environment and enhance the regeneration ability of the transplanted MSCs^26^. Therefore, CCR3A was used to stimulate pulp regeneration by transplantation of MDPSCs in the aged dog teeth model.

MDPSCs are not directly involved in pulp regeneration but can induce pulp regeneration by secreting trophic factors to elicit migration and proliferation and inhibit apoptosis of endogenous MSCs^27^. PDLSCs as representative stem cells migrating from the surrounding tissues through the apical foramen into the root canal participate in pulp regeneration. Additionally, the decline in the proliferative and migratory potential of aged PDLSCs has also been demonstrated^28^. This prompted us to investigate the biochemical properties of the PDLSCs as representative stem cells from the surrounding tissues. First, we evaluated the *in vivo* enhancement effect of CCR3A on pulp regeneration in aged dog teeth. To elucidate the mechanism of CCR3A, the rescue effect of CCR3A in the senescent HPDLCs induced by para-Cresol (*p*-Cresol) was evaluated by analyzing cell size, proliferation, and senescence marker expression. Furthermore, reduced CCL11 expression by CCR3A and its rejuvenating effects on the migratory, angiogenesis and neurite outgrowth activities were examined.

## Results

### CCR3A treatment stimulates pulp regeneration in the aged dog teeth

**Figure 1:**
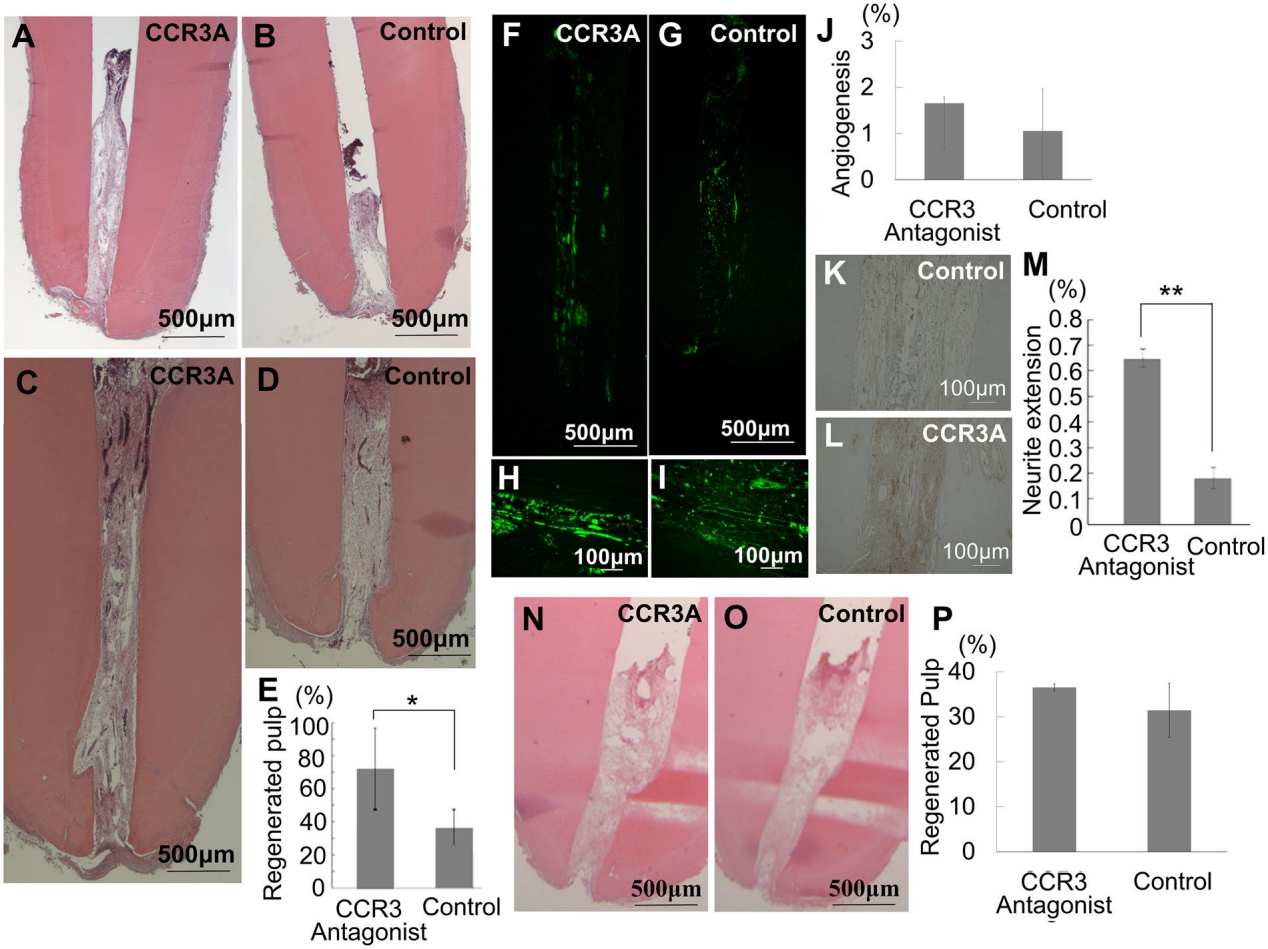
Stimulated pulp tissue regeneration and neurite extension after transplantation of mobilized dental pulp stem cells (MDPSCs) with CCR3 antagonist (CCR3A) in pulpectomized aged dog teeth (5 to 6-year-old). (**A,B**) The regenerated pulp tissue on day 14 and **(C,D)** on day 60 after transplantation. (**E,P**) Ratio of newly regenerated area to root canal area on day 60. (**F–J**) Neovasculization by BS-1 lectin staining and (**K–M**) Reinnervation by PGP 9.5 immunostaining on day 60. (**J**,**M**) Ratio of positively stained area in the regenerated pulp tissue by morphometric analyses. (**A**–**M**) In aged dog teeth. (**N–P**) In the young teeth (8 to 12-month-old). Note no difference in the amount of regenerated pulp tissue between CCR3A together with MDPSC and control MDPSC only. (**A–D**, **N,O**) Hematoxylin and eosin staining. (**E**,**J**,**M**,**P**) Data are expressed as mean ± SD (n = 3). *p < 0.05, **p < 0.01.

Histological analysis of the microenvironment of aged teeth showed chronic inflammation in a form of fibrosis in periapical tissue compared to young dog teeth (Supplementary Fig. S1)^29^ which might be a reason for the decline of pulp regeneration^9^. Thus, to investigate the enhanced regenerative capacity of CCR3A on pulp regeneration in the aged teeth, MDPSCs were transplanted together with CCR3A into the root canal after pulpectomy. After 14 and 60 days, CCR3A significantly increased the amount of pulp tissue regeneration in comparison with control non-treated (Fig. 1A–E). There are undifferentiated mesenchymal cells and immature extracellular matrix in the upper part of regenerated pulp tissue on day 14. On the other hand, the well-innervated regenerated pulp tissue is completely filled in the root canal and covered by regenerated dentin matrix in the crown part of the root canal on day 60. A larger amount of dentin was also observed along the dentinal wall on day 60 compared with on day 14 in the regenerated pulp tissue which is promoted by CCR3A (Fig. 1C,D). In the regenerated pulp tissue and periapical tissue, capillary densities did not show any difference between CCR3A treatment and control (Fig. 1F–J). However, CCR3A treatment demonstrated significantly higher neurite outgrowth compared to control (Fig. 1K–M). On the other hand, MDPSCs transplanted with CCR3A demonstrated no difference in the amount of regenerated pulp tissue compared to control in the young teeth (Fig. 1N–P).

### The optimal condition of *p*-Cresol for cellular senescence and expression of CCL11 in HPDLCs

**Figure 2:**
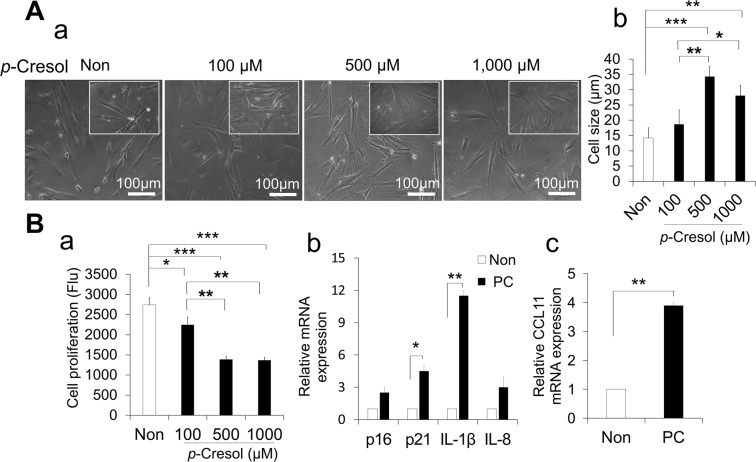
Effect of *p*-Cresol (PC) on cellular senescence in human periodontal ligament cells (HPDLCs). (**A**) (a) Morphological changes in HPDLCs after treatment with *p*-Cresol (100, 500 and 1,000 µM) for 72 h. Representative images are shown from one out of three independent experiments. (b) Determination of cell size. Values represent mean ± SD. *p < 0.05, **p < 0.01 and ***p < 0.001. (**B)** (a) After treatment with *p*-Cresol (100, 500 and 1,000 µM) for 72 h, proliferation was assessed using PrestoBlue cell viability reagents. Values represent mean ± SD (n = 3). *p < 0.05, **p < 0.01, ***p < 0.001. (b) After treatment with *p*-Cresol (500 µM) for 72 h, senescence associated markers (*p16, p21, IL-1β, and IL-8*) were assessed by RT-PCR. Values represent mean ± SD *(*n = 3). *p < 0.05, and **p < 0.01 versus non-treated. (c) Relative expression of *CCL11* mRNA after treatment with *p*-Cresol (PC) (n = 3). **p < 0.01.

To induce cellular senescence in HPDLCs, we optimized the condition of different concentrations of *p*-Cresol (100, 500 and 1,000 µM) at different duration points (24, 48 and 72 h). Treatment with *p*-Cresol at a concentration of 500 and 1,000 µM for 72 h significantly increased the cell size compared with control (p < 0.001 and p < 0.01, respectively) and with 100 µM (p < 0.01 and 0.05, respectively). There was no significant difference between 500 and 1,000 µM (Fig. 2Aa,b). PrestoBlue cell viability assay showed that 500 and 1,000 µM of *p*-Cresol after 72 h significantly decreased the proliferative capacity compared with control (p < 0.001) and with 100 µM (p < 0.01). There was no significant difference between 500 and 1,000 µM (Fig. 2Ba). The proliferation rate at different duration points (24, 48 and 72 h) has been provided at Supplementary Fig. S2, indicating that the significantly higher effect of *p*-Cresol was observed at 72 h. Therefore, *p*-Cresol at 500 µM for 72 h was considered to be optimum to induce cellular senescence. Furthermore, RT-PCR results showed that *p*-Cresol treatment at the optimal conditions significantly increased the expression of senescence-associated markers, *p21* and *IL-1β* (Fig. 2Bb). The HPDLCs treated with *p*-Cresol significantly increased expression of *CCL11* (Fig. 2Bc), indicating that CCL11 expression was increased in the senescence.

### CCR3A rescues *p*-Cresol senescence effect in HPDLCs

**Figure 3:**
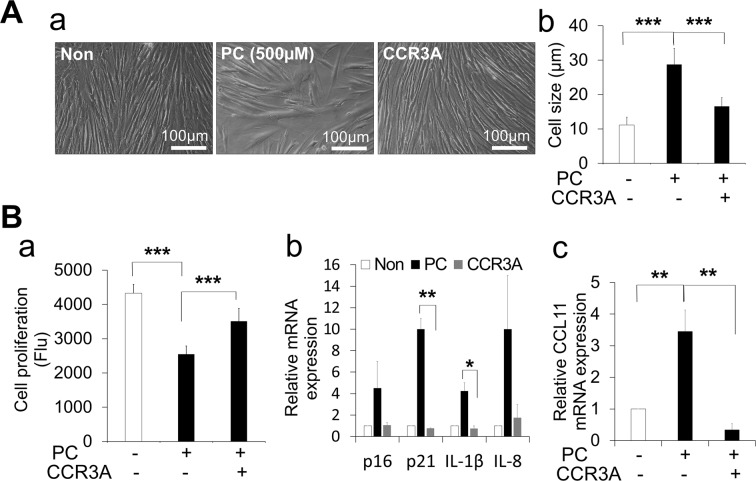
CCR3 antagonist (CCR3A) (1,000 ng/mL) rescues *p*-Cresol (PC, 500 µM for 72 h)-induced cellular senescence in human periodontal ligament cells (HPDLCs). (**A)** (a) Morphological changes of HPDLCs; non-treated, *p*-Cresol-treated, and CCR3A-treated for 3 h before *p*-Cresol exposure. Representative images are shown from one out of three independent experiments. (b) Determination of cell size. Values represent mean ± SD. ***p < 0.001. (**B)** (a) Proliferation capacity was quantified using PrestoBlue cell viability reagent. ***p < 0.001. (b) RT-PCR showed that CCR3A inhibited the increase of senescence markers, *p21 and IL-1β*. *p < 0.05, **p < 0.01. (c) HPDLCs treated with CCR3A decreased expression of *CCL11* mRNA following *p*-Cresol exposure. **p < 0.01. All values represent mean ± SD (n = 3).

To investigate the protective effect of CCR3A on cellular senescence, cell size, proliferation activity, and senescent marker expression were examined in *p*-Cresol exposed HPDLCs. Treatment with CCR3A demonstrated morphologically similar in cell shape to non-exposed control and prevented the increase in cell size (Fig. 3Aa,b). Also, the PrestoBlue cell viability assay showed that CCR3A treatment significantly inhibited the decrease in proliferation activity caused by *p*-Cresol exposure (Fig. 3Ba). Furthermore, RT-PCR analysis demonstrated that CCR3A treatment inhibited the increased expression of senescence markers, *p21 and IL-1β* by *p*-Cresol (Fig. 3Bb). Taken together, these findings suggest that CCR3A protects HPDLCs against *p*-Cresol-mediated cellular senescence.

### CCR3A decreased expression of *CCL11* and increased anti-inflammatory markers

**Figure 4:**
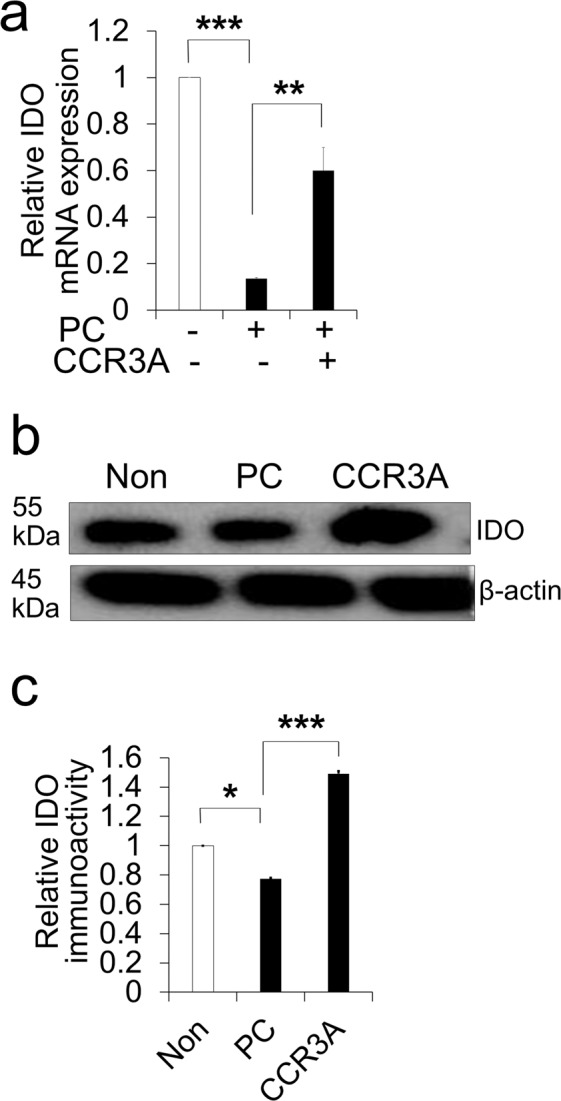
Human periodontal ligament cells (HPDLCs) treated with CCR3 antagonist (1,000 ng/mL) increased an anti-inflammatory marker. (**A**) Relative expression of *IDO* in HPDLCs by RT-PCR analysis. **p < 0.01 and ***p < 0.001. (n = 3) (**B**) Representative western blot analysis showing IDO protein expression in HPDLCs; non-treated, *p*-Cresol-treated (PC, 500 µM) for 72 h, and CCR3A-treated for 3 h before *p*-Cresol exposure cropped from different parts of the same gel. The full-length blotted membrane is available in Supplementary Fig. S3. (**C**) The quantitative analyses of IDO immunoblot. *p < 0.05, ***p < 0.001. (n = 3).

RT-PCR demonstrated that CCR3A inhibited the increase of the *CCL11* gene expression in HPDLCs after *p*-Cresol exposure (Fig. 3Bc). Furthermore, CCR3A treatment could increase the expression of gene and protein of an anti-inflammatory/immunomodulatory marker, IDO (Fig. 4a–c). These results suggest that CCR3A may have an anti-inflammatory/immunomodulatory effect.

### CCR3A enhanced neurite extension and migratory activity but not angiogenesis

**Figure 5:**
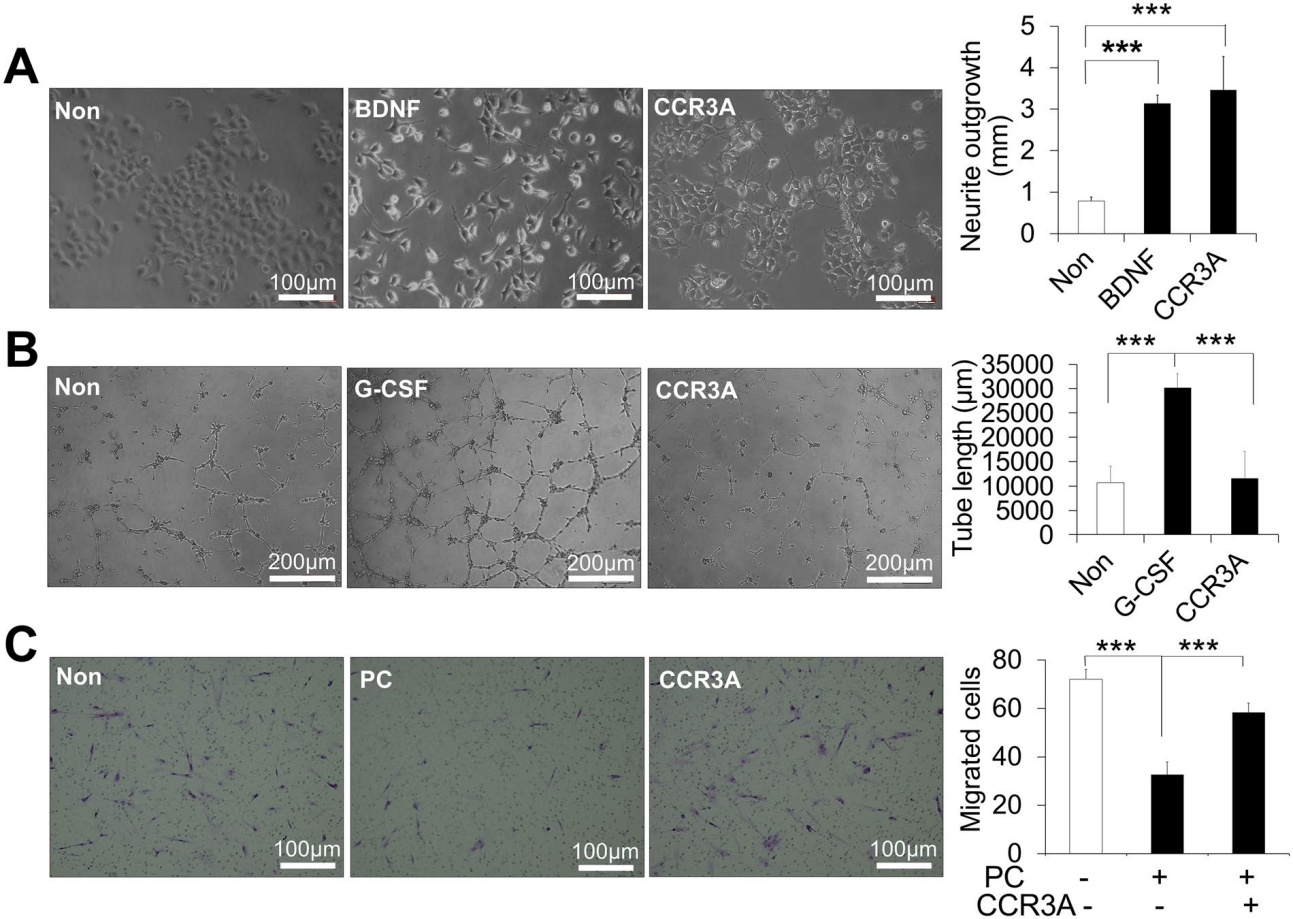
Effects of CCR3 antagonist (CCR3A, 1,000 ng/mL) on neurite extension, angiogenesis and migratory activities. (**A**) Neurite outgrowth of CCR3A on TGW cell line and neurite length of the different treatments. ***p < 0.001 (n = 3). (**B**) Angiogenesis activity of CCR3A on HUVEC cells, network formation after 5 h and the length of the tubes at 5 h. ***p < 0.001. (**C**) Migrated HPDLCs under a light microscope by transwell migration assay; non-treated, *p*-Cresol-treated (PC) and CCR3A treated before *p*-Cresol exposure and cell counts of the migrated cells. ***p < 0.001. All values represent mean ± SD (n = 3).

Next, the effects of CCR3A on neurite outgrowth, angiogenesis and migratory activities were examined. CCR3A treatment (1,000 ng/mL) for 24 h significantly increased neurite outgrowth of the TGW cell line (Fig. 5A). However, there was no effect on angiogenic tube formation in HUVEC (Fig. 5B). Moreover, the migratory activity of the *p*-Cresol exposed HPDLCs was up-regulated when treated with CCR3A compared with *p*-Cresol only (Fig. 5C).

## Discussion

The aim of the present study was to examine the effect of CCR3A on enhanced pulp regeneration in the aged dog teeth and to elucidate its underlining mechanism *in vitro*. With increasing age, there is an increase in the senescence of MSCs^30^. Senescent cells, mostly in aged cells, are not able to keep the physiological tissue repair^31^ and cause loss of the regenerative capacity^32,33^. Moreover, it has been stated that aging affects the regenerative capacity of dental pulp cells in pulp regeneration^34^, indicating in part due to senescence. We have previously demonstrated the mechanism of pulp regeneration by MDPSCs transplantation together with granulocyte-colony stimulating factor (G-CSF) in the young teeth; the transplanted MDPSCs is survived and localized by G-CSF and secrete various trophic factors which promote endogenous cell migration from the surrounding tissues of the tooth, neovascularization, re-innervation, anti-inflammation, and immunomodulation^27^. However, the regenerated pulp tissue in the aged teeth was less in comparison to the young teeth^9^. Targeting of aging mechanisms is important to reverse the aging-associated phenotypes and functions of tissue-specific stem cells. These restorative interventions hold promise for the possibilities of regenerative medicine and the treatment of many age-related diseases and dysfunctions. Chronic inflammation and cellular senescence are implicated in aging mechanisms. This knowledge has instigated us to investigate whether CCR3A with MDPSCs transplantation could protect against cellular senescence and inflammation and improve pulp regeneration in aged dog teeth. However, no need to modify cellular senescence and inflammation for pulp regeneration in young dogs in which regenerated pulp tissue was the same in CCR3A together with MDPSCs as MDPSCs alone in young dogs. It has been shown that inflammation can be beneficial and helps to stimulate regeneration and immune responses^35^. This kind of inflammation tends to end in a short period of time and be localized to an area of injury. However, aged tissues are characterized by chronic and low-grade inflammation, which play an important risk factor for diseases in the elderly people^36^. Numerous cytokines, molecular pathways, and chemokines involved in persistent chronic inflammation induce direct tissue degeneration and cause multiple age-related diseases^36^. Regeneration in aged tissues is declined due to resident stem cell senescence^32,37^, chronic inflammation^38^, decreased migration activity^39^. Angiogenesis/vaculogenesis^40^ and neurogenesis^41^ are also decreased. Modulating dental pulp cells by a variety of cytokines and growth factors can improve their regenerative capacity^42^. Consistency, our *in vivo* results demonstrated that the amount of regenerated pulp tissues was significantly increased by transplantation of MDPSCs together with CCR3A, as a stimulating factor. Suggesting that CCR3A might be involved in the rejuvenation and enhanced migration activity of resident stem cells, inhibition of inflammation, increased angiogenesis and neurogenesis in aged teeth.

Another common feature of age-related pathologies is the accumulation of senescent cells in several tissues of humans and animals^43^. The senescent state of stem cells causes impaired of their function and tissue regenerative capacity^44^. It is reported that *p*-Cresol promotes cellular senescence and inhibits proliferation through cell cycle arrest^45^. Lee et al. 2018 demonstrated that *p*-Cresol could increase the expression of the pro-senescence protein, p21^46^. Consistency, the optimized condition of *p*-Cresol at 500 µM after 72 hours could induce senescence, indicated by increased cell size, decreased proliferation activity and increased senescence markers *p21* and *IL-1β*. Moreover, CCR3A could decrease the senescence markers, *p21* and *IL-1β* and restore the proliferation activity of the senescent cells. These results suggested the role of CCR3A in the rejuvenation of resident endogenous cells in pulp regeneration in aged teeth.

Eotaxin-1/CCL11 is a chemokine involved in the recruitment of inflammatory cells into inflammatory sites through CCR3 signaling. Further studies were performed in asthma^47^, and bone resorptive inflammation^48^. As well, eotaxin-1/CCL11 is associated with the aging process, neurogenesis and neurodegeneration, being able to influence neural progenitor cells, and microglia^24^. In addition to its function in the immune response, CCL11 acts as a biological marker in pulpitis^25^. A recent study showed that aged nerves associated with chronic inflammation exhibited an increased level of CCL11^49^. Consistently, our *in vitro* senescence model induced by *p*-Cresol showed significantly higher expression of CCL11. The above-mentioned data suggest that CCL11 acts as a chronic inflammatory biomarker and therefore it hinders tissue regeneration. Thus, blocking the chemokine receptor CCR3 to prevent CCL11/CCR3 signaling is a promising therapeutic strategy to enhance aged pulp regeneration. Recently, CCR3A (R321) has been shown to have the potential for the treatment of human eosinophilic inflammation^50^. Moreover, our recent results demonstrated that the administration of CCL11 neutralizing antibody stimulated pulp regeneration and significantly decreased the blood level of CCL11 in an ectopic tooth root model of the aged mice. The underlying mechanism was explained due to improve the M1/M2 ratio and reduce the number of M1 macrophages in the regenerated pulp^51^. It has recently been clarified that macrophages are important for tissue remodeling, and especially, M2 macrophages have been reported to be involved in the creation of an environment for regenerative and repair reactions^52^. A study demonstrated that inhibition of inflammation by Acetylsalicylic acid enhanced peripheral nerve regeneration and decreased blood level of CCL11 in old mice^49^. Consistency, in the current *in vitro* results demonstrated a decreased inflammatory marker, CCL11 and increased an immunosuppressor marker, IDO by CCR3A treatment, suggesting its effect as anti-inflammation/immunosuppression in aged teeth.

Cell migration, angiogenesis, and neurite extension are playing a pivotal role during pulp tissue regeneration^53^. Moreover, the present study showed that CCR3A treatment increased neurite outgrowth *in vivo* and *in vitro*. The migration activity was also enhanced in the *in vitro* senescence model. These results suggested the effect of CCR3A on enhanced neurogenesis and migration in aged teeth. However, CCR3A did not affect angiogenesis *in vivo* and *in vitro*. CCR3 activation is reported to be essential for *in vivo* angiogenesis in a preclinical model of age-related macular degeneration and neutralization by CCR3 antibodies inhibited the tube formation of primary human circulating endothelial cells *in vitro*^54^, demonstrating consistent results of our present study. Thus, another factor to use together with CCR3A is a challenge to stimulate vasculogenesis/angiogenesis. Our collective findings support the concept that the treatment of CCR3A together with transplantation of MDPSCs can be a good approach to regenerate the pulp in aged teeth. However, the examination of the molecular mechanism by which CCR3A effects in both cellular senescence and expression of CCL11 have not yet been characterized. Our further studies are to examine these molecular mechanisms which will be critical for future therapeutic targeting of CCR3A in a clinical setting.

In conclusion, our study demonstrated that CCR3A treatment could enhance pulp regeneration in the aged dog teeth through rejuvenation and enhanced migration potential of resident endogenous cells, suppression of inflammation, and enhanced neurite extension. A suitable environment for resident endogenous cells and transplanted stem cells should be provided to enhance pulp regeneration in the aged teeth.

## Methods

### Animal care

Dogs were obtained from Kitayama Labs (Iwakuni and Ina, Japan). All experimental protocols were approved by the Animal Care and Use Committee of the National Center for Geriatrics and Gerontology, Research Institute and the Aichi Medical University (permission # 2016-5, 2017-25). All procedures and methods were performed in accordance with relevant guidelines and regulations.

### Transplantation of MDPSCs with CCR3A in dog pulpectomized teeth

Cell transplantation for pulp regeneration was performed in pulpectomized teeth as described previously^27^ with slight modification. Teeth of 5-6-year-old and 8-12-month-old dogs were used for transplantation of MDPSCs (5 × 10^5^) and G-CSF (Neutrogin, Chugai Pharmaceutical, Tokyo, Japan) in 20 μl of atelocollagen scaffold (Koken, Tokyo, Japan) with or without CCR3 antagonist (SB328437, 200 ng). The teeth were extracted on day 14 and day 60 after treatment. Histological examination of the regenerated tissue was performed in the paraffin sections (5 μm in thickness) of the teeth using a binocular microscope (Leica, M 205 FA) and its relative amount to the root canals was determined by using Leica Application Suite software (Leica, version 3.4.1). For neovascularization and innervation analyses, 5-μm-thick paraffin sections were stained by Fluorescein Griffonia (Bandeiraea) Simplicifolia Lectin 1/Fluorescein-Galanthus Nivalis (snowdrop) Lectin (BS-1 Lectin) (20 μg/mL, Vector laboratories, Inc., Youngstown) and anti-PGP9.5 (Ultra Clone) (1:10,000) respectively as previously described^27^. The ratios of newly formed capillary area and neurite extension area to the regenerated pulp area were measured respectively by Dynamic cell count BZ-HIC (Keyence, Osaka, Japan).

### Cell culture

Human periodontal ligament fibroblasts [HPDLCs, clone 3F1611, Lonza (Basel, Switzerland)] and human neuroblastoma cell line (TGW, clone JCRB 0618, Health Science Research Resources Bank, Japan) were cultured in DMEM with 10% FBS. Human umbilical vein endothelial cells (HUVEC, clone 7F3415, Lonza) were cultured in EGM2 with 5% Fetal Bovine Serum (FBS) (Life Technologies Co., USA).

### Morphometric analysis and cell proliferation assay

HPDLCs were exposed to different concentrations of *p*-Cresol (100, 500, 1,000 µM) (Sigma-Aldrich, Missouri, USA). Morphological changes were examined under an inverted microscope (Leica, 6000B-4, Leica Microsystems GmbH, Wetzlar, Germany). Briefly, individual images were obtained, and the average cell size was calculated from a minimum of 3 field images per in 3 independent wells using ImageJ software (version 1.52, imagej.nih.gov). Cell proliferation was assessed using the PrestoBlue cell viability reagent (Thermo Fisher Scientific, Japan) according to the manufacturer instructions. Briefly, HPDLCs were cultured in 96-well plates and were exposed to the different concentrations of *p*-Cresol. For proliferation assay, 90 µL of regular media was added to the well, incubated for 30 minutes at 37 °C, 5% CO_2_. Ten microliters of PrestoBlue reagent were added to the wells and incubated for 2 h. The cell numbers were measured using a spectrophotometer at 535 nm fluorescence at 24, 48, and 72 h. After determining the optimum concentration of *p*-Cresol, the rescue effect of pretreatment with CCR3A (1,000 ng/mL) was examined on cell morphology and size and proliferation capacities. The concentration of CCR3A was selected based on our preliminary data in which different concentrations of CCR3A were used (100 ng/mL, 500 ng/mL, and 1,000 ng/mL). The preliminary data showed that 1,000 ng/mL of CCR3A had a significant rescue effect against the decreased proliferation rate caused by *p*-Cresol.

### RNA isolation and quantitative reverse transcription real-time PCR

Total RNA from the different treatment of HPDLCs (non-treatment, *p*-Cresol only and pretreatment with CCR3A for 3 h before exposure to *p*-Cresol for 72 h) was extracted with TRIzol (Life Technologies, USA). First strand cDNA was generated using 1 μg of total RNA by reverse transcription using the ReverTra Ace-α kit (Toyobo, Japan) according to the manufacturer’s protocol. Reverse-transcribed products were amplified by the SYBR method using 7500 real-time PCR system (Applied Biosystems, USA) according to the manufacturer’s instruction. To examine mRNA expression of senescence markers, *p16, p21, IL-1β, IL-8* and *CCL11*, and anti-inflammatory/immunomodulatory marker, *IDO*, real-time PCR amplification of human primers were performed (Table 1). Threshold cycle number (CT) was automatically determined by ABI 7500 software and mRNA expression was normalized with β-actin.

**Table 1:** Human primers sequences used in real-time polymerase chain reaction analysis.

Primer name		Primer sequence	Size
P16	Forward Reverse	GAA GGT CCC TCA GAC ATC CCC CCC TGT AGG ACC TTC GGT GAC	94 bp
P21	Forward Reverse	GGAGACTCTCAGGGTCGAAA GGATTAGGGCTTCCTCTTGG	96 bp
IL-1β	Forward Reverse	GGCCCTAAACAGATGAAGTGCT TGCCGCCATCCAGAGG	62 bp
IL-8	Forward Reverse	TTGGCAGCCTTCCTGATTTC TCTTTAGCACTCCTTGGCAAAAC	65 bp
CCL11	Forward Reverse	ATACCCCTTCAGCGACTAGAG GCTTTGGAGTTGGAGATTTTTGG	168 bp
IDO	Forward Reverse	CAAAGGTCATGGAGATGTCC CCACCAATAGAGAGACCAGG	233 bp
β-actin	Forward Reverse	GGACTTCGAGCAAGAGATGG AGCACTGTGTTGGCGTACAG	234 bp

IL-1β, interleukin 1 beta; IL-8, interleukin 8; CCL11, C-C motif chemokine 11; IDO, indoleamine 2,3-dioxygenase.

### Western blotting analysis

Total protein was extracted from the different treatments of HPDLCs using RIPA lysis buffer (Thermo Fisher Scientific, USA). Cell lysates in sample buffer were separated on 12% SDS PAGE and transferred to a PVDF membrane for probing with antibodies. After washing, membranes were blocked with 5% skimmed milk for 2 h at room temperature then incubated with primary antibodies against IDO (1:500, Cayman Chemical, USA) and β-actin (1:1,000, Cell Signaling Technology, USA). Antigen detection was performed using a specific secondary HRP-conjugated antibody followed by exposure to Luminata Forte Western HRP Substrate. Western blotting results were detected by Amersham Imager 680 (GE Life Science, USA). The intensity of the signal obtained for each protein was quantified by densitometry using ImageJ software (version 1.52, imagej.nih.gov). Protein levels were normalized to β-actin for quantification.

### Differentiation into the neurogenic and angiogenic phenotypes

For the quantification of neurite outgrowth, the human neuroblastoma cell line (TGW) was starved overnight and then stimulated with CCR3A for 24 h. The mean neurite length was measured under the inverted microscope using ImageJ software (version 1.52, imagej.nih.gov). The same experiment was performed with 50 ng/mL Brain-derived neurotrophic factor (BDNF) (Peprotech, UK) as a positive control. To evaluate the angiogenic effect of CCR3A on HUVEC, HUVEC were seeded on Matrigel (BD Biosciences, San Jose, USA) in DMEM containing 2% FBS, 5 µg/mL heparin (Lonza), 5 µg/mL ascorbic acid (Lonza), 5 µg/mL hydrocortisone (Lonza) supplemented with or without CCR3A. DMEM containing only 2% FBS was used as a negative control and G-CSF (100 ng/mL) was used as a positive control. The mean length of networks of cords and tube-like structures was measured 5 h after cultivation under an inverted microscope (Leica, 6000B-4, Leica Microsystems GmbH, Wetzlar, Germany) using ImageJ software (version 1.52, imagej.nih.gov).

### Transwell migration assay

To determine the migratory activity in response to G-CSF, the migratory activity of pretreated HPDLCs with CCR3A following exposure to *p*-Cresol for 72 h was compared to *p*-Cresol treated only. Non-senescence HPDLCs were kept as a control. The HPDLCs were resuspended in serum-free medium and adjusted to a density of 1 × 10^6^ cells/mL. Transwell inserts (6.5-mm diameter and 8-mm pore size; Corning, Inc.) were loaded with the cell suspensions (100 µL), and 600 µL of 2% FBS supplemented with G-CSF (100 ng/mL) was added to the lower chambers. After incubation for 24 h at 37 °C, non-migrated cells were scrub-off from the surface of the insert, the attached cells remaining on the bottom of the insert were fixed with 95% Methanol and stained with 10% Giemsa stain for 15-minute. After washing, the stained cells were counted under an inverted bright-field microscope (Keyence, Osaka, Japan) at × 100 magnification. The experiment was performed in triplicate.

### Statistical analyses

All results were expressed as means ± standard deviation. Data were analyzed statistically using *t*-test or one-way analysis of variance (ANOVA) with Tukey Comparison Test as a post-test using SPSS 21.0 (IBM, Armonk, NY).

## Supplementary information

Supplementary information.

## Data Availability

All data generated or analyzed during this study are included in this published article.

## References

[1] Sadamori S (2012). Nutritional status and oral status of the elderly with dementia: A 2-year study. Gerodontology.

[2] Sadamori S, Hayashi S, Hamada T (2008). The relationships between oral status, physical and mental health, nutritional status and diet type in elderly japanese women with dementia. Gerodontology.

[3] Nakashima M, Iohara K (2017). Recent progress in translation from bench to a pilot clinical study on total pulp regeneration. J. Endod..

[4] Caplan AI, Correa D (2011). The msc: An injury drugstore. Cell. Stem Cell..

[5] Nurkovic J (2016). Aging of stem and progenitor cells: Mechanisms, impact on therapeutic potential, and rejuvenation. Rejuvenation Res.

[6] Narbonne P (2018). The effect of age on stem cell function and utility for therapy. Cell. Med..

[7] Lopez-Otin C, Blasco MA, Partridge L, Serrano M, Kroemer G (2013). The hallmarks of aging. Cell..

[8] Nakashima M, Iohara K (2014). Mobilized dental pulp stem cells for pulp regeneration: Initiation of clinical trial. J. Endod..

[9] Iohara K, Murakami M, Nakata K, Nakashima M (2014). Age-dependent decline in dental pulp regeneration after pulpectomy in dogs. Exp. Gerontol..

[10] Pinchi V, Forestieri AL, Calvitti M (2007). Thickness of the dental (radicular) cementum: A parameter for estimating age. J. Forensic Odontostomatol..

[11] Boyle M (2014). Chronic inflammation and angiogenic signaling axis impairs differentiation of dental-pulp stem cells. PLoS One.

[12] Martins-Green M, Petreaca M, Wang L (2013). Chemokines and their receptors are key players in the orchestra that regulates wound healing. Adv. Wound Care.

[13] Adar T, Shteingart S, Ben Ya’acov A, Bar-Gil Shitrit A, Goldin E (2014). From airway inflammation to inflammatory bowel disease: Eotaxin-1, a key regulator of intestinal inflammation. Clin. Immunol..

[14] Garcia-Zepeda EA (1996). Human eotaxin is a specific chemoattractant for eosinophil cells and provides a new mechanism to explain tissue eosinophilia. Nat. Med.

[15] Bartels J (1996). Human dermal fibroblasts express eotaxin: Molecular cloning, mrna expression, and identification of eotaxin sequence variants. Biochem. Biophys. Res. Commun..

[16] Bostrom EA (2015). Increased eotaxin and mcp-1 levels in serum from individuals with periodontitis and in human gingival fibroblasts exposed to pro-inflammatory cytokines. Plos One.

[17] Alblowi J (2013). Chemokine expression is upregulated in chondrocytes in diabetic fracture healing. Bone.

[18] Kitaura M (1996). Molecular cloning of human eotaxin, an eosinophil-selective cc chemokine, and identification of a specific eosinophil eotaxin receptor, cc chemokine receptor 3. J. Biol. Chem..

[19] Ye J, Kohli LL, Stone MJ (2000). Characterization of binding between the chemokine eotaxin and peptides derived from the chemokine receptor ccr3. J. Biol. Chem..

[20] Menzies-Gow A (2002). Eotaxin (ccl11) and eotaxin-2 (ccl24) induce recruitment of eosinophils, basophils, neutrophils, and macrophages as well as features of early- and late-phase allergic reactions following cutaneous injection in human atopic and nonatopic volunteers. J. Immunol..

[21] Paplinska M (2012). Expression of eotaxins in the material from nasal brushing in asthma, allergic rhinitis and copd patients. Cytokine.

[22] Owczarek W (2010). Analysis of eotaxin 1/ccl11, eotaxin 2/ccl24 and eotaxin 3/ccl26 expression in lesional and non-lesional skin of patients with atopic dermatitis. Cytokine.

[23] Kokkonen H (2010). Up-regulation of cytokines and chemokines predates the onset of rheumatoid arthritis. Arthritis Rheum..

[24] Hoefer J (2017). The “aging factor” eotaxin-1 (ccl11) is detectable in transfusion blood products and increases with the donor’s age. Front. Aging Neurosci..

[25] Abd-Elmeguid A (2013). Osteocalcin expression in pulp inflammation. J. Endod..

[26] Segers VF, Lee RT (2011). Biomaterials to enhance stem cell function in the heart. Circ. Res..

[27] Iohara K (2013). A novel combinatorial therapy with pulp stem cells and granulocyte colony-stimulating factor for total pulp regeneration. Stem Cells Transl Med.

[28] Zhang J (2012). The effect of aging on the pluripotential capacity and regenerative potential of human periodontal ligament stem cells. Biomaterials.

[29] Wynn TA, Ramalingam TR (2012). Mechanisms of fibrosis: Therapeutic translation for fibrotic disease. Nat. Med.

[30] Alves H (2012). A mesenchymal stromal cell gene signature for donor age. PLoS One.

[31] Rafi MA, Alavi A (2017). Debate on human aging and lifespan. BioImpacts: BI.

[32] van Deursen JM (2014). The role of senescent cells in ageing. Nature.

[33] Campisi J, d’Adda di Fagagna F (2007). Cellular senescence: When bad things happen to good cells. Nat. Rev. Mol. Cell Biol..

[34] Iezzi I, Pagella P, Mattioli-Belmonte M, Mitsiadis TA (2019). The effects of ageing on dental pulp stem cells, the tooth longevity elixir. Eur Cell Mater.

[35] Okada S, Ogata T (2016). Inflammation and regeneration in cross-organs. Inflamm Regen.

[36] Franceschi C, Campisi J (2014). Chronic inflammation (inflammaging) and its potential contribution to age-associated diseases. J. Gerontol. A Biol. Sci. Med. Sci..

[37] Paez-Ribes M, Gonzalez-Gualda E, Doherty GJ, Munoz-Espin D (2019). Targeting senescent cells in translational medicine. EMBO molecular medicine.

[38] Josephson AM (2019). Age-related inflammation triggers skeletal stem/progenitor cell dysfunction. Proceedings of the National Academy of Sciences.

[39] Brown AD, Close GL, Sharples AP, Stewart CE (2017). Murine myoblast migration: Influence of replicative ageing and nutrition. Biogerontology.

[40] Sadoun E, Reed MJ (2003). Impaired angiogenesis in aging is associated with alterations in vessel density, matrix composition, inflammatory response, and growth factor expression. J. Histochem. Cytochem..

[41] Mathews KJ (2017). Evidence for reduced neurogenesis in the aging human hippocampus despite stable stem cell markers. Aging cell.

[42] Nakashima M, Iohara K, Murakami M (2013). Dental pulp stem cells and regeneration. Endodontic Topics.

[43] Herbig U, Ferreira M, Condel L, Carey D, Sedivy JM (2006). Cellular senescence in aging primates. Science (New York, N.Y.).

[44] Neves J, Demaria M, Campisi J, Jasper H (2015). Of flies, mice, and men: Evolutionarily conserved tissue damage responses and aging. Developmental cell.

[45] Chang MC (2014). P-cresol affects reactive oxygen species generation, cell cycle arrest, cytotoxicity and inflammation/atherosclerosis-related modulators production in endothelial cells and mononuclear cells. Plos One.

[46] Lee JH, Yun CW, Hur J, Lee SH (2018). Fucoidan rescues p-cresol-induced cellular senescence in mesenchymal stem cells via fak-akt-twist axis. Mar Drugs.

[47] Wu D (2014). Ccl11 as a potential diagnostic marker for asthma?. J. Asthma.

[48] Kindstedt E (2017). Ccl11, a novel mediator of inflammatory bone resorption. Sci Rep.

[49] Buttner R (2018). Inflammaging impairs peripheral nerve maintenance and regeneration. Aging cell.

[50] Pease JE, Williams TJ (2019). Tipping the balance: A biased nanobody antagonist of ccr3 with potential for the treatment of eosinophilic inflammation. J. Allergy Clin. Immunol..

[51] Hayashi Y, Kawamura R, Nishimatsu SI, Fukuta O, Nakashima M (2019). Stem cell-induced pulp regeneration can be enhanced by administration of ccl11-neutralizing antibody in the ectopic tooth transplantation model in the aged mice. Rejuvenation Res.

[52] Spiller KL, Koh TJ (2017). Macrophage-based therapeutic strategies in regenerative medicine. Adv Drug Deliv Rev.

[53] Nakashima M (2017). Pulp regeneration by transplantation of dental pulp stem cells in pulpitis: A pilot clinical study. Stem Cell. Res. Ther.

[54] Takeda A (2009). Ccr3 is a target for age-related macular degeneration diagnosis and therapy. Nature.

