# Supplementary material for: CCR3 antagonist protects against induced cellular senescence and promotes rejuvenation in periodontal ligament cells for stimulating pulp regeneration in the aged dog
Source: Sci Rep. 2020 May 25;10:8631. doi: 10.1038/s41598-020-65301-9 (PMC7248074; doi:10.1038/s41598-020-65301-9)
Supplement: Supplementary file 1 — Supplementary information. [file 41598_2020_65301_MOESM1_ESM.pdf]

Supplemental Materials

CCR3 antagonist protects against induced cellular senescence and promotes rejuvenation in periodontal ligament cells for stimulating pulp regeneration in the aged dog

Mohammed Zayed[1, 2], Koichiro Iohara[1], Hideto Watanabe[3], Misako Nakashima[1, 4*]

[1]Department of Stem Cell Biology and Regenerative Medicine, National Center for Geriatrics and Gerontology, Research Institute, Obu, Aichi, 474-8511 Japan.

[2]Department of Animal Surgery, College of Veterinary Medicine, South Valley University, Qena, 83523 Egypt.

[3]Institute for Molecular Science of Medicine, Aichi Medical University, Nagakute, Aichi, 480-1195 Japan.

[4]Aeras Bio Inc., Air Water Group, Kobe, Hyogo, 650-0047 Japan

[*]**Correspondence to:** Misako Nakashima, PhD, Department of Stem Cell Biology and Regenerative Medicine, National Center for Geriatrics and Gerontology, 7-430 Morioka, Obu, Aichi 474-8511, Japan. Telephone: +81-562-44-5651 ext. 5063; Fax: +81-562-46-8684; E-mail: misako@ncgg.go.jp

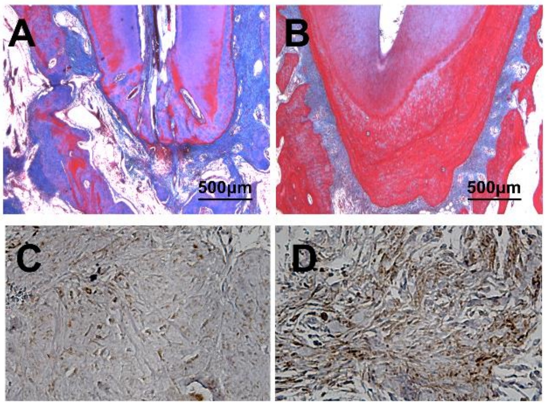

Supplementary figure S1

Aging of tooth is characterized by stenosis and fibrosis of periodontal ligament, widening of cementum, constriction of the apical region and mineralization of pulp tissue, affecting resident stem cell function and homeostasis of tooth. **A, C.** Young tooth Periapical area **B, D**. Aged tooth periapical area. **A, B.** Masson trichrome staining. **C, D.** Immunohistology staining of Vimentin.

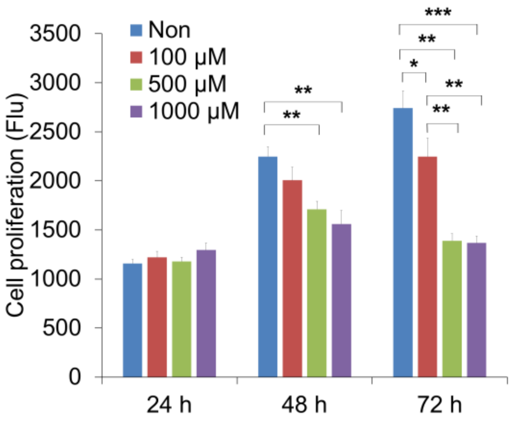

Supplementary figure S2

Effect of *p*-Cresol (PC) on cellular senescence in human periodontal ligament cells (HPLCs). After treatment with *p*-Cresol (100, 500 and 1000 μM), proliferation rate was assessed at 24, 48 and 72 h using PrestoBlue cell viability reagents. Values represent mean ± SD (n = 3). *p < 0.05, **p< 0.01 ***p < 0.001.

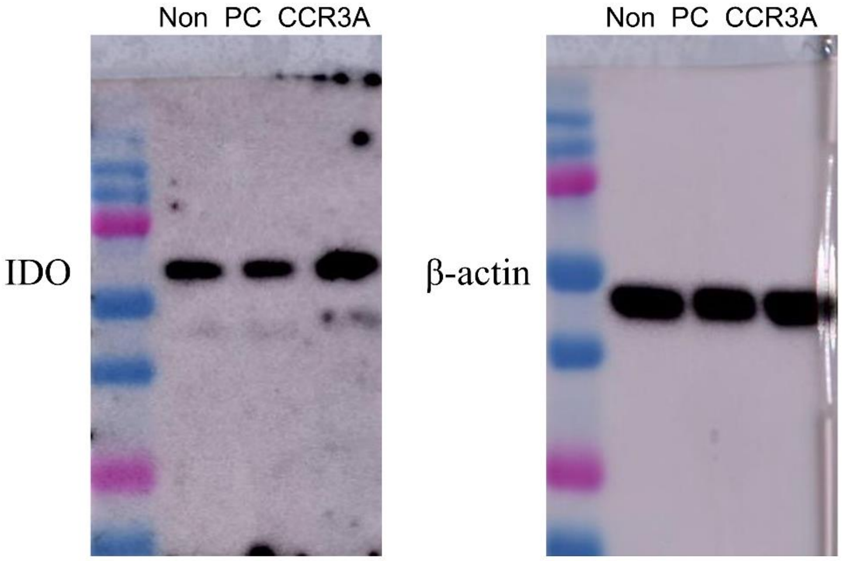

Supplementary figure S3

Original western blot membrane results showing IDO protein expression in HPDLCs; non-treated, *p*-Cresol-treated (PC,500 µM) for 72 h, and CCR3A-treated (1,000 ng/mL) before *p*-Cresol exposure.
